# Intragenic DNA methylation in buccal epithelial cells and intellectual functioning in a paediatric cohort of males with fragile X

**DOI:** 10.1038/s41598-018-21990-x

**Published:** 2018-02-26

**Authors:** Marta Arpone, Emma K. Baker, Lesley Bretherton, Minh Bui, Xin Li, Simon Whitaker, Cheryl Dissanayake, Jonathan Cohen, Chriselle Hickerton, Carolyn Rogers, Mike Field, Justine Elliott, Solange M. Aliaga, Ling Ling, David Francis, Stephen J. C. Hearps, Matthew F. Hunter, David J. Amor, David E. Godler

**Affiliations:** 1 Faculty of Medicine, Dentistry and Health Sciences, Department of Paediatrics, University of Melbourne, Parkville, VIC Australia; 2 Cyto-Molecular Diagnostics Research, Murdoch Children’s Research Institute, Royal Children’s Hospital, Parkville, VIC Australia; 3 Child Neuropsychology, Murdoch Children’s Research Institute, Royal Children’s Hospital, Parkville, VIC Australia; 4 Melbourne School of Psychological Sciences, University of Melbourne, Melbourne, VIC Australia; 5 Centre for Epidemiology and Biostatistics, Melbourne School of Population and Global Health, University of Melbourne, Melbourne, VIC Australia; 6 School of Human and Health Science, University of Huddersfield, Queensgate, Huddersfield United Kingdom; 7 Olga Tennison Autism Research Centre, La Trobe University, Melbourne, VIC Australia; 8 Fragile X Alliance Inc, North Caulfield, VIC, Australia and Centre for Developmental Disability Health Victoria, Monash University, Dandenong, VIC Australia; 9 Genetics of Learning Disability Service (GOLD service), Hunter Genetics, Newcastle, NSW Australia; 10 Victorian Clinical Genetics Services and Murdoch Children’s Research Institute, Royal Children’s Hospital, Parkville, VIC Australia; 11 Centre for Diagnosis and Treatment of Fragile X Syndrome, INTA University of Chile, Santiago, Chile; 12 Monash Genetics, Monash Health, Melbourne, VIC, Australia and Department of Paediatrics, Monash University, Melbourne, VIC Australia

## Abstract

Increased intragenic DNA methylation of the Fragile X Related Epigenetic Element 2 (FREE2) in blood has been correlated with lower intellectual functioning in females with fragile X syndrome (FXS). This study explored these relationships in a paediatric cohort of males with FXS using Buccal Epithelial Cells (BEC). BEC were collected from 25 males with FXS, aged 3 to 17 years and 19 age-matched male controls without FXS. Methylation of 9 CpG sites within the FREE2 region was examined using the EpiTYPER approach. Full Scale IQ (FSIQ) scores of males with FXS were corrected for floor effect using the Whitaker and Gordon (WG) extrapolation method. Compared to controls, children with FXS had significant higher methylation levels for all CpG sites examined (p < 3.3 × 10^−7^), and within the FXS group, lower FSIQ (WG corrected) was associated with higher levels of DNA methylation, with the strongest relationship found for CpG sites within *FMR1* intron 1 (p < 5.6 × 10^−5^). Applying the WG method to the FXS cohort unmasked significant epi-genotype-phenotype relationships. These results extend previous evidence in blood to BEC and demonstrate FREE2 DNA methylation to be a sensitive epigenetic biomarker significantly associated with the variability in intellectual functioning in FXS.

## Introduction

Fragile X syndrome (FXS) is the most common single gene cause of inherited intellectual disability (ID), estimated to occur in ~1 in 4,000–5,000 males and in ~1 in 5,000–8,000 females^1,2^. The presence of >200 CGG repeats, downstream of the *FMR1* promoter, is termed full mutation (FM) and is the most frequent cause of FXS^2^. FM alleles are associated with increased DNA methylation of the regulatory regions proximal to the CGG expansion. These regions include the *FMR1* CpG island and the Fragile X Related Epigenetic Element 2 (FREE2) region at the *FMR1* exon 1/intron 1 boundary, located 5′ and 3′ of the expansion, respectively^3,4^. Higher levels of methylation of the CpG island and the FREE2 region have been correlated with a lower expression of Fragile X Mental Retardation Protein (FMRP) in blood^4–6^ and lower IQ scores^6,7^. Lower IQ scores have also been associated with reduced FMRP levels^6,8–10^: FMRP has a critical function in synaptic plasticity and brain development and its loss is thought to cause the FXS neurodevelopmental phenotype^11,12^. Furthermore, in females with *FMR1* premutation (PM) expansions, between 55 and 199 CGG repeats, higher levels of DNA methylation at FREE2 have been associated with neuro-cognitive and psychiatric phenotypes^13–15^. Together, these studies link the epigenotype with the type and severity of the phenotype in fragile X related disorders in women.

An important limitation of these epigenotype-phenotype studies is the lack of accurate measures of cognitive functioning in children with ID, whose IQ scores are often at ‘floor’ level when derived using standardized assessments^9,16–20^. The ‘floor effect’ occurs when most data points, for example scale scores (SS) and standardized composite scores such as the Full Scale IQ (FSIQ), correspond to the minimal score obtainable on the test, and cluster at the lower end of the distribution. Most intelligence tests, including the Wechsler Scales^21,22^, do not measure IQ below 40. In these tests, subtest items in the lower range are few and limited in task difficulty. Moreover, a wide range of raw scores for each subtest corresponds to ‘floored’ minimum SS of 1. This leads to less sensitive and reliable measurement of heterogeneity in intellectual abilities among individuals functioning in the extremely low range. Tackling this sensitivity issue has major implications clinically, where it is necessary to tailor interventions based on specific individual strengths and weaknesses. This is also of importance in the research context where IQ scores impact study design, patient stratification, outcome evaluation, and associations with other outcomes such as molecular and clinical biomarkers.

This study compared the levels of FREE2 DNA methylation in buccal epithelial cells (BEC) in a paediatric cohort of males with FXS and an age-matched control group of male children without FXS. The Whitaker and Gordon (WG) extrapolation method^18^ was used to correct for the ‘floor effect’ that affects the Wechsler intelligence test scores obtained by children with FXS. The relationships between FREE2 DNA methylation in BEC and WG-corrected FSIQ scores were then examined in the FXS cohort. In particular, as part of this analysis, we were interested to compare methylation levels at intronic FREE2 sites with exonic FREE2 sites, as in females with FM, methylation at intronic FREE2 sites has showed much stronger correlation with IQ scores^7^.

## Methods

### Participants with FXS

The primary sources of participant recruitment were Victorian Clinical Genetics Services, Monash Genetics and Hunter Genetics, and support organizations including the Fragile X Association of Australia and Fragile X Alliance Inc. All participants underwent fragile X genetic testing prior to recruitment. Presence of FM alleles was confirmed using Southern Blots and/or AmplideX PCR sizing [Asuragen, Inc., Austin, TX, USA]. From our Australian nation-wide FREE FX study, we selected male children for whom BEC FREE2 methylation results were available and whose intellectual abilities were assessed with an age appropriate Wechsler Intelligence test. Twenty-five male children fulfilled these criteria and are included in this study. Age at participation (e.g. chronological age at time of cognitive assessment and BEC collection) varied between 3.3 and 16.9 years (Median = 6.4 years; Interquartile range (IQR) = 6.4). Participants were biologically unrelated except for two sets of brothers (2^a^ and 15^a^, who were born from consanguineous parents, and 4^b^ and 12^b^; Table 1). *FMR1* CGG size for 25 participants with FXS is reported in Table 1. Seventeen (68%) participants carried exclusively a FM allele and eight had PM/FM size mosaicism, defined as the presence of different CGG repeat sizes (some PM and some FM) in different cells from one individual. For PM alleles, FREE2 methylation is typically within the range observed in controls with <40 CGG repeats^23^. Therefore, participants with PM/FM size mosaicism are expected to have lower levels of FREE2 DNA methylation compared to participants who have a *FMR1* CGG expansion exclusively in the FM range. Participants’ ethnicity can be found in the Supplementary Table S1.

**Table 1:** Age and CGG size for all 25 males with FXS.

ID	Age at assessment	CGG size	*FMR1* expansion category
1	3.32	210–630	FM
2^a^	3.36	>200^#^	FM
3	3.37	>200^#^	FM
4^b^	3.46	103; >200^#^	PM/FM
5	4.13	75; 150–813	PM/FM
6	4.41	113; 163–540	PM/FM
7	4.47	90; 200–420	PM/FM
8	4.97	672–1025	FM
9	5.01	322	FM
10	5.34	280–413	FM
11	5.44	343–890	FM
12^b^	5.99	590, 823–1263	FM
13	6.41	385	FM
14	6.97	303–803	FM
15^a^	7.87	173–516	PM/FM
16	7.97	377–970	FM
17	8.12	503–570	FM
18	8.60	280–480	FM
19	10.91	197; 313–463	PM/FM
20	12.80	170; 171; 197; >200^#^	PM/FM
21	13.37	110; 180–1400	PM/FM
22	13.42	200–433	FM
23	14.30	477–1263	FM
24	14.41	523	FM
25	16.93	360–760	FM

Note: same letter next to ID indicate relatedness: 2^a^ and 15^a^ are brothers born from consanguineous parents; 4^b^ and 12^b^ are brothers born from unrelated parents.^#^FM alleles were detected with AmplideX that cannot accurately size alleles with >200 CGG repeats.

### Control participants without FXS

Among all control participants who took part in the FREE FX study, we selected control male children (n = 19) for whom BEC FREE2 methylation results were available. Participants’ age, at BEC collection, varied between 2.0 and 15.7 years (Median = 7.6 years; IQR = 6.6). Six control children were recruited by contacting women who had previously participated in fragile X carrier screening studies within the Murdoch Children’s Research Institute (MCRI) and resulted to have normal size *FMR1* alleles (<45 CGG repeats). Three of these control children underwent formal cognitive testing at MCRI where their re-identifiable BEC samples were collected by the research team. The other three control children had their cheek brush samples collected at home by their parents. The remaining 13 controls were recruited through flyers distributed within MCRI and the Royal Children’s Hospital, Melbourne, inviting employees to participate with their children (4 to 17 years), in the study. Employees were asked to collect their children’s samples at home. All BEC samples collected by parents were returned with a reply paid envelope to the research team in an irreversibly anonymised fashion. Exclusion criteria for all control participants can be found as Supplementary note.

Parents/guardians of participants in the FXS and control groups, for whom re-identifiable BEC samples were collected, provided signed informed consent. No written consent was sought from control families who exclusively provided anonymous samples; as explained in the information statement, returning the samples constituted consent to participate in the control arm of the research project. All study procedures were in accordance with the Declaration of Helsinki and approved by the Royal Children’s Hospital Human Research Ethics Committee (Single Site: HREC 34227A and HREC 33066F; Multi site HREC: HREC/13/RCHM/24).

### Molecular Analyses

Up to four BEC samples were collected per participant using the Master Amp Buccal Swab Brush kit (Epicentre Technologies, Madison, WI, USA). Each swab was inspected independently for blood contamination by at least two staff members at the time of sample collection, and/or at the time of sample receipt prior to processing. Two out of all collected brushes had confirmed blood contamination and were discarded. DNA was extracted from the remaining BEC samples using the NucleoSpin^®^ Tissue genomic DNA extraction kit (Machery-Nagel, Duren, Germany) and then transferred to fresh 96-well plates to be treated with sodium bisulphite as previously described^23,24^. Each BEC DNA sample was bisulphite converted using the EZ DNA Methylation-Gold^TM^ kit in two separate reactions, with each conversion analysed in duplicate reactions using the EpiTYPER system.

To explore differences in DNA methylation levels between the exonic and intronic regions within FREE2, DNA methylation of five CpG units was analysed, comprising overall 9 CpG sites: CpG1 and CpG2 located within *FMR1* exon 1 and CpG6/7, CpG8/9 and CpG10-12 within intron 1. Notably, the methylation levels of CpG6/7, CpG8/9 and CpG10-12 could not be analysed separately at single CpG resolution. This is because the fragments generated through base-specific T cleavage, for each of the CpG site within each CpG unit, have the same mass^4^. Therefore, as described previously^4^, the fragments cannot be distinguished after the mass cleave reaction. This precludes single CpG resolution by the matrix assisted laser desorption/ionization-time of flight mass spectrometry (MALDI-TOF MS) analysis, which relies on the mass size ratio of the cleaved products to provide quantitative methylation estimates for each CpG site. A summary measure for each CpG unit was determined as the mean of two or more methylation output ratio (MOR) measurements from the EpiTYPER system per BEC DNA sample. The analytical sensitivity of the EpiTYPER assay was 0.10 MOR, as previously defined^4^.

DNA extracted from all irreversibly anonymized control buccal samples (n = 16) was also used for CGG size testing with PCR, as previously described^25^. All these controls’ BEC samples had CGG repeats length within the normal range (CGG < 45). Re-identifiable BEC samples (n = 3) were not tested for CGG size. However, it is extremely unlikely that these children had an expansion or mutation of the *FMR1* gene, considering all the exclusion criteria (see Supplementary Note) and their mothers’ <45 CGG size, which is considered stable upon intergenerational transmission^26^.

### Neuropsychological Assessments of Participants with FXS

Depending on the chronological age of the participant, one of two standardized measures of cognitive functioning was administered to obtain FSIQ: the Wechsler Preschool and Primary Scale of Intelligence, Third Edition Australian Standardised Edition (WPPSI-III Australian)^21^ (≥3 years and <7 years; n = 14) and the Wechsler Intelligence Scale for Children, Fourth Edition Australian Standardised Edition (WISC-IV Australian)^22^ (≥7 years; n = 11). Three cognitive outcome measures were used for epigenotype-phenotype analyses: (i) standardized FSIQ (FSIQ), obtained according to the standardized procedures outlined in the WPPSI-III and WISC-IV manuals; (ii) ‘standardized FSIQ + default FSIQ’ (dFSIQ) which include the FSIQ, as defined in (i), plus default minimum FSIQ of 40 assigned to those with an invalid FSIQ score, as they could not be derived according to the standardized procedures. As described in the “invalidating composite scores” section in the respective test manuals, when a participant obtained raw scores of zero, for example on 2 of the 3 subtests (including potential subtest substitution) composing the Verbal Comprehension Index on the WISC-IV or the Verbal IQ on the WPPSI-III, no FSIQ could be derived; and lastly (iii) ‘WG corrected FSIQ’ (cFSIQ), calculated using the WG extrapolation method^18^. Whitaker and Gordon^18^ illustrated the extent of the floor effect on IQ scores obtained by adolescents in special education who were assessed with the WISC-IV UK Edition. In this previous study, all raw scores that obtained a SS of 1 were re-examined. In order to calculate FSIQ scores corrected for the floor effect, the best fit equations between raw scores and scale scores (SS) and between sum of scale scores (SSS) and FSIQ were determined, by using the raw score to SS and the SSS to FSIQ conversion data available in the published test manual. This study applied the same WG method^18^ to the WISC-IV Australian Edition and the WPPSI-III Australian Edition to obtain corrected FSIQ for all participants having any subtest SS equal to 1. A brief explanation of the application of Whitaker and Gordon extrapolation method to the WISC-IV Australian Edition is provided in the Supplementary method.

### Statistical Analyses

Descriptive statistics were performed to characterize participants’ age at time of assessment, FREE2 DNA methylation and FSIQ; the Shapiro-Wilk test was used to examine the normality of these variables’ data distribution. Comparisons of FREE2 DNA methylation output ratio (MOR) for each CpG unit (MOR for CpG1, CpG2, CpG6/7, CpG8/9 and CpG10-12) between (i) the FXS (FM + PM/FM) and the control group, (ii) the FM only and the control group, (iii) the PM/FM mosaics and the control group and (iv) the PM/FM mosaic and the FM only group were performed with non-parametric Mann-Whitney U tests. Non-parametric ROC curve analysis was used to evaluate the ability of each CpG unit MOR to discriminate between groups based on CGG size category (PM/FM vs FM; PM/FM vs control; FM vs control). For the FXS group, Spearman correlation analyses were run to determine the relationships between each of the 5 FREE2 CpG unit MOR. Regression analysis was used to assess whether each of three types of FSIQ, (namely FSIQ, dFSIQ, and cFSIQ), or each of the 5 FREE2 CpG unit methylation outcomes depended on age. The same method was used for examining the inter-relationships between each of the five BEC FREE2 DNA methylation variables (predictor) and each FSIQ, adjusted for age whenever the relationship between age and FSIQ was found to be significant. Least square regression was used as: (i) an estimation method; (ii) a model diagnostic for outlier observations. If outliers were present, we used robust regression to down-weight the effect of outliers on estimated parameters. If the robust regression could not be performed due to the small sample size, we conducted inference using least square method with outliers excluded. In the preliminary analyses, we also fitted the linear random effects model to the data to adjust for relatedness. This model was tested against the ordinary linear regression, which assumes that all data are independent, using the likelihood-ratio test. These relationships were not significant, suggesting that adjusting for relatedness was not required in this study. To adjust for multiple testing, we used false discovery rate (FDR). All analyses were performed using Stata statistical software (version 13). Lastly, one participant (ID 20; Table 1) with PM/FM size mosaicism, was identified to have levels of FREE2 DNA methylation that were within the control range. Further analyses were conducted with this outlier participant removed from the dataset. Inter-group comparisons of mean FREE2 DNA methylation levels were performed with Student’s two-sample t-tests and the best CpG unit MOR to discriminate between groups, based on CGG size category, was ascertained with non-parametric ROC curve analysis.

### Data availability

The datasets generated and analysed during the current study are available from the corresponding author on reasonable request.

## Results

**Figure 1:**
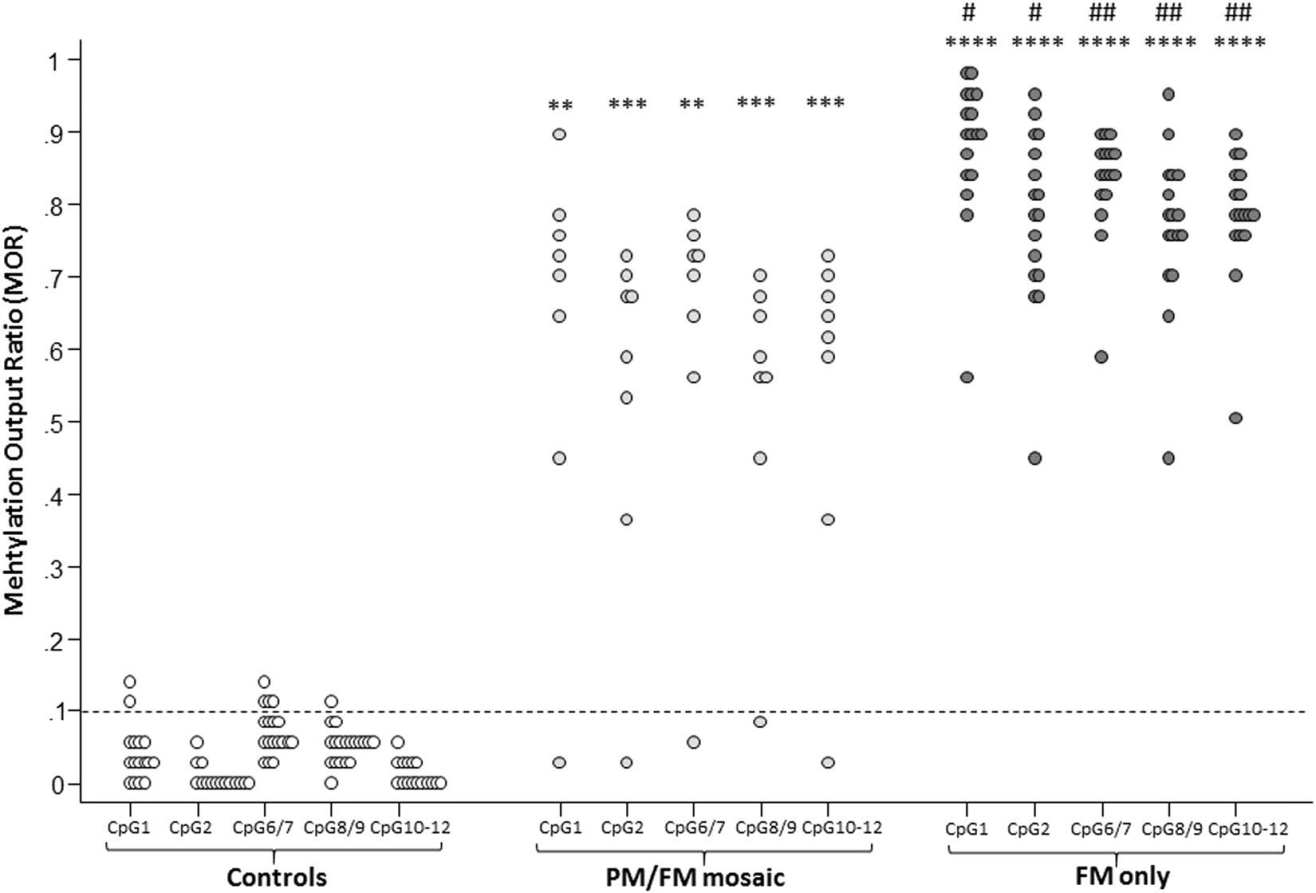
Inter-group comparison of FREE2 DNA methylation in buccal epithelial cells. Controls: control participants with normal *FMR1* CGG size (<45 CGG); PM/FM: participants with *FMR1* PM/FM (premutation/full mutation) CGG size mosaicism; FM: participants with *FMR1* FM CGG expansion only. Note: the parallel black broken lines at 0.1 MOR represents the analytical sensitivity of the EpiTYPER assay, as previously defined^4^; ****p < 1.3 × 10^−6^ for the comparison between controls and participants with FM expansion (see Supplementary Table S5); ***p < 0.0005 for the comparison between controls and participants with PM/FM size mosaicism (see Supplementary Table S6); **p < 0.001 for the comparison between controls and participants with PM/FM size mosaicism (see Supplementary Table S6); ^##^p < 0.001 for the comparison between participants with PM/FM size mosaicism and participants with FM only (see Supplementary Table S3); ^#^p < 0.005 for the comparison between participants with PM/FM size mosaicism and participants with FM only (see Supplementary Table S3).

In males with FXS, median MORs for all FREE2 CpG units were greater than 70%, with the lowest median MOR for CpG2 being 71.5% and the highest for CpG1 being 86.0% (Table 2). No participant had 100% methylation for any of the FREE2 CpG units investigated. One participant (ID 20; Table 1; Fig. 1) had less than 10% methylation across all FREE2 CpG units. For the entire FXS cohort, MORs of all CpG units were highly correlated (*r*_*s*_ > 0.793; p < 3.8 × 10^−6^) with each other (see Supplementary Table S2). There were no significant relationships between age (predictor) and DNA methylation levels (outcome) for all FREE2 CpG units examined. FXS intra-groups comparison revealed that participants with PM/FM size mosaicism, compared to participants with FM only, had significantly lower levels of methylation for all CpG units (Fig. 1; see Supplementary Table S3). However, MOR for CpG10-12 was the best discriminant between the ‘PM/FM mosaic’ and ‘FM only’ groups, with an area under the curve (AUC) of 0.949, the highest among all CpG units (Fig. 1; see Supplementary Table S4).

**Table 2:** Comparison of FREE2 DNA methylation in buccal epithelial cells between the FXS and control group.

	FXS				Controls				FXS vs Controls	
	n	Median (IQR)	Mean (SD)+	Min-Max	n	Median (IQR)	Mean (SD)	Min-Max	p-value*	p-value**
MOR CpG1	25	0.860 (0.170)	0.825 (0.134)	0.020–0.975	16	0.027 (0.038)	0.039 (0.038)	0.005–0.130	**3**.**2** **×** **10**^**−7**^	**1**.**2** **×** **10**^**−23**^
MOR CpG2	25	0.715 (0.150)	0.730 (0.144)	0.015–0.965	15	0.000 (0.010)	0.009 (0.015)	0.000–0.055	**2**.**9** **×** **10**^**−7**^	**8**.**6** **×** **10**^**−21**^
MOR CpG6/7	24	0.804 (0.139)	0.787 (0.097)	0.060–0.900	18	0.064 (0.045)	0.070 (0.028)	0.025–0.130	**2**.**9** **×** **10**^**−7**^	**5**.**0** **×** **10**^**−28**^
MOR CpG8/9	25	0.750 (0.135)	0.716 (0.129)	0.080–0.950	19	0.050 (0.020)	0.052 (0.023)	0.010–0.105	**1**.**3** **×** **10**^**−7**^	**4**.**5** **×** **10**^**−24**^
MOR CpG10-12	25	0.760 (0.115)	0.735 (0.126)	0.040–0.890	16	0.010 (0.006)	0.015 (0.010)	0.005–0.045	**2**.**2** **×** **10**^**−7**^	**1**.**2** **×** **10**^**−23**^

Abbreviations: n, number of participants’ samples from which MALDI-TOF MS results were available; IQR, interquartile range; SD, standard deviation; Min – Max = minimum and maximum values; MOR, methylation output ratio; Adjusted p-values (for multiple testing using FDR) computed using *Mann-Whitney test to compare the median between the two groups and **two-sample t-test to compare the mean between the two groups, where + mean and SD in the FXS group are computed with one outlier excluded; p-values less than 0.05 are highlighted in bold.

Participants in the control group had median FREE2 MOR, which were less than 7% for all CpG units (Fig. 1; Table 2). As expected, significant differences were found in FREE2 MOR between the FXS and the control group, with the former having significantly (p < 3.3 × 10^−7^) higher methylation levels for all CpG units than the latter (Table 2). The stratification of the FXS group based on CGG size category showed that there was no overlap in MOR values between the control and FM group for any of the CpG units (Fig. 1); AUC for all units was equal to 1. Non-parametric comparison of median MOR between these two groups showed highly significant differences (p < 1.3 × 10^−6^) (see Supplementary Table S5). Although one participant (ID 20) with PM/FM mosaicism had MOR values within the controls’ range, the remaining 7 participants with size mosaicism had MOR values well above the controls (Fig. 1). Overall, the PM/FM group, compared to the controls, had significantly higher levels of methylation for all CpG units (p < 0.0008) (see Supplementary Table S6). Nevertheless, the intronic CpG10-12 MOR discriminated best (AUC = 0.992) the PM/FM from the control group (Fig. 1; see Supplementary Table S4). The exclusion of the outlier (ID 20), with PM/FM size mosaicism and normal FREE2 DNA methylation, did not have any substantial impact on the results for the inter-group comparison analyses involving the PM/FM and the FM only groups. It was evident that the significant differences found between these two groups, when all 25 participants with FXS were included, were not due to the presence of the outlier. In fact, despite the exclusion of this participant, the PM/FM group had, for all FREE2 CpG units analysed, mean methylation levels that were significantly lower (p < 0.005) than the FM only group (see Supplementary Table S3). As expected, the inter-group comparison between the combined FXS (PM/FM size mosaicism plus FM only) and the control groups, after the exclusion of participant 20, demonstrated lower FDR adjusted p-values (p < 9 × 10^−21^) than the results of the analyses which included all participants with FXS (p < 3.3 × 10^−7^) (Table 2). The ROC curve analyses without the outlier showed that the exclusion of this participant did not have any relevant effect on the results and further corroborated that, based on AUC estimates, the intronic CpG10-12 unit best discriminates the PM/FM from the FM group (Fig. 1; see Supplementary Table S4).

FSIQ scores were obtained from 48% of participants with FXS (9 with FM and 3 with PM/FM mosaicism) with 10 of these 12 FSIQ scores being greater than 40. These 12 FSIQ were normally distributed (Table 3). The FSIQ of the remaining 13 participants (13/25; 52%) were considered invalid as they could not be derived according to standardized procedures, due to the preponderance of raw scores of 0. When the 13 invalid scores were substituted with default FSIQ of 40 and added to the 12 FSIQ, the overall distribution of these 25 dFSIQ was skewed towards the floor of 40 (Table 3). Conversely, following the application of the WG method to all SS of 1 obtained by the participants, newly corrected cFSIQ were generated for 19 participants, including all 13 who had invalid standardized composite scores. The remaining six FSIQ (ranging from 52 to 73) were unchanged after the WG correction. Overall, the 25 cFSIQ ranged from 6 to 73 (Table 3), had a normal distribution (Table 3) and produced a complete FSIQ dataset, extending well below the floor level of 40 to a corrected score of 6.

**Table 3:** Descriptive statistics results of FSIQ scores.

	n	Median	IQR	Min-Max	Shapiro-Wilk test p* value
Standardized FSIQ (FSIQ)	12	53	15	40–73	0.266
Standardized + default FSIQ (dFSIQ)	25	40	12	40–73	**<0**.**001**
WG corrected FSIQ (cFSIQ)	25	43	26	6–73	0.295

Abbreviations: n, number of participants; WG cFSIQ = FSIQ corrected with the Whitaker and Gordon method; IQR, interquartile range; Min – Max = minimum and maximum values; MOR, methylation output ratio; FSIQ, Full Scale IQ; *p-value for testing normal distribution of a variable using Shapiro-Wilk test; values less than 0.05 are considered significant, indicate a not normal distribution and are highlighted in bold.

Regression analyses did not show significant relationships between age (predictor) and FSIQ (regression coefficient (β) = −0.902; p = 0.298), nor between age (predictor) and dFSIQ (β = −0.50; p = 0.132). However, the relationship between age and cFSIQ was statistically significant (β = −3.612, p < 0.001), with decrease in cFSIQ associated with increased age. Therefore, in subsequent analyses involving a relationship with this variable the adjustment for age was included.

**Figure 2:**
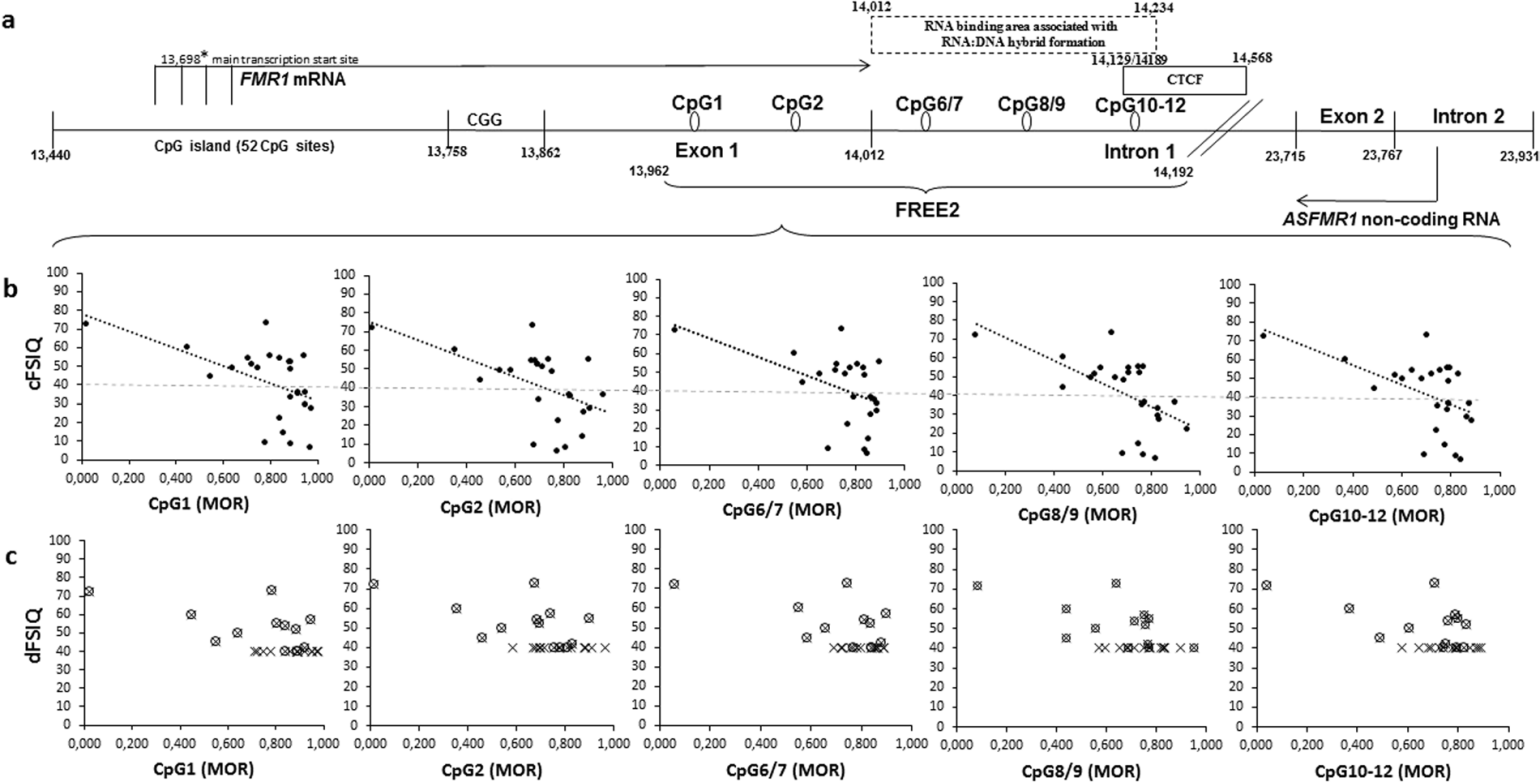
Relationships between intragenic DNA methylation in buccal epithelial cells and FSIQ. (**a**) Organization of the Xq27.3 sequence encompassing specific FREE2 CpG sites at the *FMR1* exon 1/intron 1 boundary (GenBank L29074 L38501) targeted by MALDI-TOF MS methylation analysis. The CTCF box indicates 5′ CTCF binding sites from UCSF Chip-Seq which overlap with FREE2 CpG10-12; the RNA:DNA hybrid box indicates locations of forward and reverse primers used in ChiRP to show formation of RNA:DNA hybrids denoted as fP(200-400) in Colak *et al*.^32^. (**b**) Relationships between corrected FSIQ, obtained through extrapolation using the Whitaker and Gordon method (cFSIQ), and FREE2 DNA methylation at the *FMR1* intron 1/exon 1 boundary, measured in buccal epithelial cells (BEC). (**c)** Relationships between FREE2 DNA methylation in BEC and standardized FSIQ (FSIQ) only (open circles) with invalid scores omitted; and dFSIQ (crosses), which include FSIQ and invalid scores included as default of FSIQ of 40. Note: black broken lines represent lines of best fit from linear regression analyses. The parallel grey broken line highlights the FSIQ of 40 as the floor of standardized FSIQ scores.

The regression analyses between the 12 FSIQ and all DNA methylation variables showed a significant relationship for only one CpG unit (CpG8/9; p = 0.042) which however became non-significant after FDR adjustment (p = 0.210) (Table 4). In contrast, the regression analyses between the 25 dFSIQ scores, where the invalid scores were included as default scores of 40, and DNA methylation variables showed significant relationships for all CpG units, even after FDR adjustment (p < 0.002; Table 4 and Fig. 2c). The cFSIQ for the 25 participants, showed stronger relationships with all CpG units (p < 5.6 × 10^−5^), with higher effect size, compared to FSIQ scores only and dFSIQ (Table 4). All relationships were in the expected direction, with increase in methylation levels associated with decrease in FSIQ scores (Fig. 2b). Furthermore, the strength of the relationships increased for the three *FMR1* intron 1 CpG units, as compared to those located within exon 1, as indicated by the estimated regression coefficients (Table 4).

**Table 4:** Relationships between FREE2 DNA methylation for each CpG unit and FSIQ in males with FXS.

MOR	FSIQ^a^				dFSIQ^b^				cFSIQ^c^			
	n	*β*	s.e	p	n	*β*	s.e	p	n	*β*	s.e	p
CpG1	11	−11.90	20.14	0.618	25	−32.25	5.87	**3**.**5** **×** **10**^**−5**^	25	−63.22	7.23	**6**.**5** **×** **10**^**−8**^
CpG2	11	−15.07	19.22	0.618	25	−30.80	7.06	**2**.**5** **×** **10**^**−5**^	25	−64.80	8.34	**2**.**4** **×** **10**^**−7**^
CpG6/7	10	−15.32	29.53	0.618	24	−38.09	6.02	**1**.**2** **×** **10**^**−5**^	24	−68.39	11.72	**1**.**1** **×** **10**^**−5**^
CpG8/9	12	−28.81	12.38	0.210	25	−32.50	8.87	**1**.**0** **×** **10**^**−3**^	25	−66.57	13.36	**5**.**5** **×** **10**^**−5**^
CpG10-12	11	−11.41	21.85	0.618	25	−33.60	7.55	**2**.**5** **×** **10**^**−4**^	25	−70.00	9.27	**2**.**5** **×** **10**^**−7**^

Abbreviation: FSIQ = standardized FSIQ; dFSIQ = Standardized + default FSIQ; cFSIQ = FSIQ corrected with the Whitaker and Gordon method; MOR, methylation output ratio; analyses conducted: ^a^Linear regression unadjusted for age; ^b^Robust regression unadjusted for age; ^c^Robust regression adjusted for age; *β*, estimated regression coefficient; s.e., standard error. Note: all p-values reported are adjusted for multiple testing (FDR); p-values less than 0.05 are highlighted in bold.

## Discussion

This study adopted the WG extrapolation method^18^ to address the floor effect impacting the standardized scale and composite IQ scores, obtained by males with FXS, on two widely used standardized intelligence tests. Through this extrapolation method we successfully attained corrected FSIQ scores for all participants. The corrected scores showed a normal distribution and demonstrated significant inter-individual variability. Importantly, this study demonstrates that in boys with FXS, increase in methylation of all five FREE2 CpG units, encompassing 9 CpG sites spanning the *FMR1* exon 1/intron 1 boundary, was significantly associated with the decrease in FSIQ corrected using the WG method.

This study also compared FREE2 DNA methylation levels in BEC between male paediatric participants with FXS and age-matched male controls without an *FMR1* expansion: children with FXS resulted to have significantly higher levels of FREE2 DNA methylation in comparison to controls. Moreover, despite the strong correlations found between the methylation levels of each CpG unit, the MOR values of CpG10-12, located within *FMR1* intron 1, performed best in discriminating PM/FM from controls and from participants with CGG expansion exclusively in the FM range.

The observed increase in BEC FREE2 DNA methylation extends previous studies where FREE2 methylation levels, associated with FM, were elevated in different tissue types, including adult and newborn blood, lymphoblasts, chorionic villi, and primary neurons from post mortem brains^4,5,7,27–30^. Notably, the FREE2 region has also been referred to via a different name – for example *FMR1* “down-stream region” as in Esanov *et al*. study^30^. The findings of elevated FREE2 methylation associated with FM alleles compared to controls, have been independently validated in FXS embryonic stem cells^31^. This was associated with abnormal histone modification and *FMR1* regulation, and may be due to the recently described formation of RNA:DNA hybrids in FXS within the intronic region of FREE2^32^ (Fig. 2a). Disruption of the interaction between the mRNA and its genomic complementary CGG-repeat portion prevented *FMR1* epigenetic silencing^32^. This is consistent with our previous studies in FM females where *FMR1* intron 1 methylation was correlated with both FMRP levels in blood and IQ assessed using standardized intelligence tests^5,7^. It is also in line with the findings of the current study in males with FXS, where the relationships between methylation levels and cFSIQ were stronger, based on regression coefficients, for the three *FMR1* intron 1 CpG units compared to the two CpG units located within exon 1 of *FMR1*.

However, in contrast to our previous study of a larger cohort of females with FM^7^, standardized FSIQ scores in males with FM showed a significant relationship for only one CpG unit (CpG8/9). This relationship was lost after adjustment for multiple testing. This suggests that the lack of significant findings for the analyses of the relationships involving FSIQ scores for only 12 participants might be due to the small sample size. Conversely, the epi-genotype-phenotype relationships using more complete dFSIQ dataset were significant for all CpG units after adjustment for multiple testing. However, the inclusion of default FSIQ of 40, as part of dFSIQ scores, led to a flat ‘floored’ pattern of data distribution (Fig. 2c). This obscured the variability in performance between the participants. In contrast, there was variability in WG scores and highly significant relationships were found between FREE2 DNA methylation and intellectual functioning when the cFSIQ obtained through the WG method was used. These results highlight that exclusion of, or using minimum scores for paediatric participants with invalid FSIQ, diminishes the ability to detect significant epi-genotype-phenotype relationships in males with FXS.

Limitations of this study are the small sample size and analysis of methylation in only one tissue type. It is also important to note that the buccal samples may have included some white blood cells in addition to BEC cells; therefore, the DNA methylation profiles obtained may represent a composite of these two cell types. A further limitation is the exclusive use of the MALDI-TOF MS EpiTYPER approach. Future studies should explore use of pyrosequencing or clonal bisulfite sequencing on BEC samples, as these techniques have been previously used to examine FREE2 methylation in other cell types^31^. These methylation analysis techniques can discriminate between a small proportion of CpG sites that cannot be discriminated using the EpiTYPER approach (CpG sites that have the same fragment weight). However, the EpiTYPER system does have major advantages over both pyrosequencing and clonal bisulfite sequencing, including much higher throughput and ability to analyse methylation across much larger regions of DNA as detailed in Tost and Gut^33^. Furthermore, a strong body of literature supports the use of the EpiTYPER system for quantitative methylation analysis in the broader epigenetics field^33^, and more specifically in the fragile X field for FREE2 methylation analysis^4,5,7,13–15,23,27^. It is important to note that an additional limitation in this study is the wide age range of the participants, which may have prevented investigations of epigenotype-phenotype relationships within specific developmental stages (for instance, early childhood vs adolescence) and has imposed limitations on the WG method.

We have found a highly significant relationship between age and cFSIQ, with lower FSIQ being associated with older age. This finding could be an epiphenomenon of the cross-sectional design, as restrospective and prospective longitudinal studies involving children with FXS have reported a relative decline of IQ scores with increasing age, even when the participants were re-assessed with the same intelligence test^34–38^. Undoubtedly however, this finding is also due to the use of the WG correction method. Whilst the strength of this method is its applicability to any standardized test, based on the transformation of raw scores to scale scores then to composite scores, a drawback is the fact that the calculations of WG corrected scores are affected by the child’s age. Thus, relationships between WG corrected scores and biomarkers need to be adjusted for age.

Other researchers have attempted to address the floor effect inherent in intelligence tests in individuals with ID^9,19,20^. These studies obtained, with permission from the publisher, the original standardization sample subtest raw score descriptive statistics, and used these data to calculate new normalized scale and IQ scores by applying a z-score transformation to each individual’s scores. Hessl *et al*.^9^ used this method in a study involving males and females with FXS, and related WISC-III normalized subtests scale scores to the percentage of FMRP positive cells in blood. The newly recalculated WISC-III normalized subtests scale scores no longer showed a floor effect, were normally distributed, and extended more than three standard deviations below the mean compared to standard scores. This method of using deviation from population is one strategy to improve IQ measurement in invididuals with ID. However, unlike the WG method, it relies on the availability of the descriptive statistics from the normative sample, which are not readily available to researchers and clinicians.

Whilst it is hoped that intelligence tests will be refined to be more sensitive to variation in cognition, this study illustrates that the WG method is a feasible methodological approach that could benefit studies involving children with significant cognitive impairments whose IQ scores are subject to the floor effect. Our approach, although requiring further validation, could help evaluate the associations between molecular factors and clinical phenotypes in other neurodevelopmental disorders. Moreover, this study presents the novel use of BEC as tissue of choice for FREE2 DNA methylation analysis in children with FXS. The use of BEC can provide a less invasive and less expensive alternative to venous blood for FREE2 methylation studies aiming to (i) examine gene-environment interactions, (ii) stratify participants in clinical trials, and (iii) study the natural history of fragile X-related disorders. In addition, BEC, in contrast with blood cells, share the same ectodermal embryological origin as brain cells, potentially making them a more informative surrogate tissue than blood^39^ for epigenotype-phenotype studies of neuropsychiatric disorders.

In summary, this study demonstrated that through the WG method we obtained a complete FSIQ dataset for a paediatric cohort of males with FXS, as compared to the standardized FSIQ scores. The use of this method has also uncovered significant epi-genotype-phenotype relationships by examining methylation of BEC DNA in a paediatric cohort of males with FXS. These relationships were not evident when standardized FSIQ scores were used. Importantly, BEC samples as used in this study is a more convenient sample type for clinicians as it does not require specialised staff, shipment or storage. The results extend on a now substantial body of evidence describing FREE2 DNA methylation as a sensitive epigenetic biomarker significantly associated with the variability in intellectual functioning in FXS. The Whitaker and Gordon extrapolation method effectively addressed the issue of IQ floor effect in children with FXS by unravelling difference in cognitive performance, with implication for other neurodevelopmental conditions associated with intellectual disability.

## Electronic supplementary material

Supplementary data
