# Supplementary material for: Intragenic DNA methylation in buccal epithelial cells and intellectual functioning in a paediatric cohort of males with fragile X
Source: Sci Rep. 2018 Feb 26;8:3644. doi: 10.1038/s41598-018-21990-x (PMC5827525; doi:10.1038/s41598-018-21990-x)
Supplement: Supplementary file 1 — Supplementary data [file 41598_2018_21990_MOESM1_ESM.docx]

**Supplementary Data**

**Intragenic DNA methylation in buccal epithelial cells and intellectual functioning in a paediatric cohort of males with fragile X**

Marta Arpone*^1,2,3^, Emma K. Baker^2^, Lesley Bretherton^1,3,4^, Minh Bui^5^, Xin Li^2^, Simon Whitaker^6^, Cheryl Dissanayake^7^, Jonathan Cohen^8^, Chriselle Hickerton^2^, Carolyn Rogers^9^, Mike Field^9^, Justine Elliott^10^, Solange M. Aliaga^1,2,11^, Ling Ling^2^, David Francis^10^, Stephen J.C. Hearps^3^, Matthew F. Hunter^12^, David J. Amor^†1,10^, David E. Godler^†1,2^.

^1^ Faculty of Medicine, Dentistry and Health Sciences, Department of Paediatrics, University of Melbourne, Parkville, VIC, Australia.

^2^ Cyto-Molecular Diagnostics Research, Murdoch Children’s Research Institute, Royal Children's Hospital, Parkville, VIC, Australia.
^3^ Child Neuropsychology, Murdoch Children’s Research Institute, Royal Children's Hospital, Parkville, VIC, Australia.
^4^ Melbourne School of Psychological Sciences, University of Melbourne, Melbourne, VIC, Australia.

^5^ Centre for Epidemiology and Biostatistics, Melbourne School of Population and Global Health, University of Melbourne, Melbourne, VIC, Australia.

^6^ School of Human and Health Science, University of Huddersfield, Queensgate, Huddersfield, United Kingdom.

^7^Olga Tennison Autism Research Centre, La Trobe University, Melbourne, Australia.

^8^ Fragile X Alliance Inc, North Caulfield, VIC, Australia and Centre for Developmental Disability Health Victoria, Monash University, Dandenong, VIC, Australia.

^9^ Genetics of Learning Disability Service (GOLD service), Hunter Genetics, Newcastle, NSW, Australia.

^10^Victorian Clinical Genetics Services and Murdoch Children’s Research Institute, Royal Children's Hospital, Parkville, VIC, Australia.

^11^Centre for Diagnosis and Treatment of Fragile X Syndrome, INTA University of Chile, Santiago, Chile.

^12^ Monash Genetics, Monash Health, Melbourne, VIC, Australia and Department of Paediatrics, Monash University, Melbourne, VIC, Australia.

^†^These authors contributed equally to this work

*Corresponding author:

Marta Arpone

The Murdoch Children’s Research Institute

The Royal Children’s Hospital

50 Flemington Rd, Parkville, Victoria, 3052 AUS

Telephone: + 61 3 8341 6245

[marta.arpone@mcri.edu.au](mailto:marta.arpone@mcri.edu.au)

**Supplementary Table S1: Ethnicity of participants with FXS**

**Supplementary Note: Exclusion criteria for all control participants**

**Supplementary Method: Example of Whitaker and Gordon extrapolation method applied to the WISC-IV Australian Edition**

**Supplementary Table S2: Correlations analyses of FREE2 MOR between each CpG unit in males with FXS**

**Supplementary Table S3: Comparison of FREE2 DNA methylation in buccal epithelial cells between males with PM/FM mosaicism and males with FM only**

**Supplementary Table S4: Area under the curve (AUC) for each CpG unit**

**Supplementary Table S5: FREE2 DNA methylation in buccal epithelial cells of males with FM only and comparison with control males**

**Supplementary Table S6: FREE2 DNA methylation in buccal epithelial cells of males with PM/FM mosaicism and comparison with control males**

| **Supplementary Table S1: Ethnicity of participants with FXS** | | | |
| --- | --- | --- | --- |
|  |  | n | % |
| Australian of European Descent | | 19 | 76 |
| Middle Eastern | | 2 | 8 |
| Southern Asian | | 1 | 4 |
| Southern-East Asian | | 1 | 4 |
| Australian of European Descent/ Southern Asian | | 1 | 4 |
| Australian of European Descent/ Polynesian | | 1 | 4 |

| **Supplementary Note: Exclusion criteria for all control participants** |
| --- |
| Exclusion criteria for all control participants included:   - age > 17 years - presence of a genetic, neurological, psychiatric or psychopathological disorder, intellectual disability, developmental delay or autism - cognitive difficulties and behavioural problems concerning the parents/guardian - family history of intellectual impairments, developmental delay, autism or early and late - onset *FMR1* related disorders. |
|  |

| **Supplementary Method: Example of Whitaker and Gordon extrapolation method applied to the WISC-IV Australian Edition.** |
| --- |
| Briefly, the WISC-IV Australian manual indicates that for the Digit Span subtest (age: 13:4 years) all raw scores from 0 to 8 are equivalent to a standard SS of 1 and that the transformation of raw scores (>7) to SS up to 10, follows an increase of one SS per increment of one raw score. Therefore, the calculated regression equation for the relationships between these raw scores and SS is equivalent to y = x - 7 (where y = SS; x = raw score). Under the assumption that this relationship hypothetically continues below the minimum SS of 1, an individual who obtained a raw score of 3, was assigned a WG corrected SS of – 4. This process was repeated for each subtest. The corrected SS were summed together to obtain new adjusted SSS, which were used to obtain newly WG corrected FSIQ (cFSIQ), using the SSS-FSIQ transformation data available in the manual or by extrapolating the SSS-FSIQ relationships below the minimum standard SSS. |
|  |

| **Supplementary Table S2: Correlations analyses of FREE2 MOR between each CpG unit in males with FXS** | | | | |
| --- | --- | --- | --- | --- |
|  | MOR CpG1 | MOR CpG2 | MOR CpG6/7 | MOR CpG8/9 |
| MOR CpG2 | *r_s_* = 0.795;  **p = 2.1x10^-6^** | - | - | - |
| MOR CpG6/7 | *r_s_* = 0.887;  **p = 7.9x10^-9^** | *r_s_* = 0.802;  **p = 2.5x10^-6^** | - | - |
| MOR CpG8/9 | *r_s_* = 0.833;  **p = 2.4x10^-7^** | *r_s_* = 0.843;  **p = 1.3x10^-7^** | *r_s_* = 0.794;  **p = 3.7x10^-6^** | - |
| MOR CpG10-12 | *r_s_* = 0.877;  **p = 3.5x10^-9^** | *r_s_* = 0.825;  **p = 4.0x10^-7^** | *r_s_* = 0.838;  **p = 3.3x10^-7^** | *r_s_* = 0.854;  **p = 5.6x10^-8^** |
| Note: males with FXS (n = 25) include participants with FM only (n = 17) and participants with PM/FM size mosaicism (n = 8). Abbreviations: *r_s_* = rho Spearman correlation coefficient; p = p- value of Spearman correlation; p < 0.05 are highlighted in bold. | | | | |

| **Supplementary Table S3: Comparison of FREE2 DNA methylation in buccal epithelial cells between males with PM/FM mosaicism and males with FM only** | | | | | | | | | | | | | | | | |
| --- | --- | --- | --- | --- | --- | --- | --- | --- | --- | --- | --- | --- | --- | --- | --- | --- |
|  |  | PM/FM | | | |  | FM | | | | | |  | PM/FM vs FM | | |
|  | n | | Median (IQR) | Mean (SD)+ | Min-Max | | |  | n | Median (IQR) | Mean (SD) | Min-Max | | |  | p-value* p-value** |
| MOR CpG1 | 8 | | 0.718 (0.220) | 0.706 (0.136) | 0.020-0.890 | | |  | 17 | 0.890 (0.105) | 0.874 (0.100) | 0.550 - 0.975 | | |  | **0.0015 0.0036** |
| MOR CpG2 | 8 | | 0.627 (0.240) | 0.606 (0.127) | 0.015-0.715 | | |  | 17 | 0.780 (0.180) | 0.781 (0.120) | 0.460 - 0.965 | | |  | **0.0015 0.0042** |
| MOR CpG6/7 | 8 | | 0.705 (0.140) | 0.698 (0.077) | 0.060-0.783 | | |  | 16 | 0.845 (0.070) | 0.827 (0.078) | 0.585 - 0.900 | | |  | **0.0008 0.0035** |
| MOR CpG8/9 | 8 | | 0.583 (0.172) | 0.601 (0.092) | 0.080-0.710 | | |  | 17 | 0.770 (0.080) | 0.763 (0.111) | 0.440 - 0.950 | | |  | **0.0009 0.0036** |
| MOR CpG10-12 | 8 | | 0.625 (0.218) | 0.614 (0.120) | 0.040-0.725 | | |  | 17 | 0.795 (0.075) | 0.785 (0.091) | 0.490 - 0.890 | | |  | **0.0004 0.0035** |
| Abbreviations: n, number of participants’ samples from which MALDI-TOF MS results were available; IQR, interquartile range; SD, standard deviation; Min – Max = minimum and maximum values; MOR, methylation output ratio; Adjusted p-values (for multiple testing using FDR) computed using *Mann-Whitney test to compare the median between the two groups and **two-sample t-test to compare the mean between the two groups, where +mean and SD in the PM/FM group computed with one outlier excluded; p-values less than 0.05 are highlighted in bold. | | | | | | | | | | | | | | | | |

**Supplementary Table S4: Area under the curve (AUC) for each CpG unit.**

|  | Outlier present | | | | | | Outlier excluded | | |
| --- | --- | --- | --- | --- | --- | --- | --- | --- | --- |
|  | PM/FM versus FM | |  | PM/FM versus controls | |  | PM/FM versus FM | | PM/FM versus controls |
|  | Estimate | 95% CI |  | Estimate | 95% CI |  | Estimate | 95% CI | Estimate |
| MOR CpG1 | 0.901 | 0.76-1.00 |  | 0.930 | 0.79-1.00 |  | 0.887 | 0.73-1.00 | 1.00 |
| MOR CpG2 | 0.901 | 0.78-1.00 |  | 0.975 | 0.92-1.00 |  | 0.887 | 0.75-1.00 | 1.00 |
| MOR CpG6/7 | 0.930 | 0.83-1.00 |  | 0.924 | 0.77-1.00 |  | 0.920 | 0.80-1.00 | 1.00 |
| MOR CpG8/9 | 0.919 | 0.81-1.00 |  | 0.984 | 0.95-1.00 |  | 0.908 | 0.78-1.00 | 1.00 |
| MOR CpG10-12 | 0.949 | 0.86-1.00 |  | 0.992 | 0.97-1.00 |  | 0.941 | 0.84-1.00 | 1.00 |

Note: The FM and control groups, as well as PM/FM and control groups if one outlier (participant ID: 20) was removed in the PM/FM group, were completely separated for each CpG unit, see Figure 1.

| **Supplementary Table S5: FREE2 DNA methylation in buccal epithelial cells of males with FM only and comparison with control males** | | | | | | | | | | |
| --- | --- | --- | --- | --- | --- | --- | --- | --- | --- | --- |
|  | FM | | |  |  | Controls | | |  | FM vs Controls |
|  | n | Median (IQR) | Min-Max |  |  | n | Median (IQR) | Min-Max |  | p-value |
| MOR CpG1 | 17 | 0.890 (0.105) | 0.550-0.975 |  |  | 16 | 0.027 (0.038) | 0.005-0.130 |  | **9.4x10^-7^** |
| MOR CpG2 | 17 | 0.780 (0.180) | 0.460-0.965 |  |  | 15 | 0.000 (0.010) | 0.000-0.055 |  | **1.2x10^-6^** |
| MOR CpG6/7 | 16 | 0.845 (0.070) | 0.585-0.900 |  |  | 18 | 0.064 (0.045) | 0.025-0.130 |  | **6.7x10^-7^** |
| MOR CpG8/9 | 17 | 0.770 (0.080) | 0.440-0.950 |  |  | 19 | 0.050 (0.020) | 0.010-0.105 |  | **3.0x10^-7^** |
| MOR CpG10-12 | 17 | 0.795 (0.075) | 0.490-0.890 |  |  | 16 | 0.010 (0.006) | 0.005-0.045 |  | **7.4x10^-7^** |
| Abbreviations: n, number of participants’ samples from which MALDI-TOF MS results were available; IQR, interquartile range; Min – Max = minimum and maximum values; MOR, methylation output ratio; p-value = FDR adjusted p-value of Mann-Whitney test; p-values < 0.05 are highlighted in bold. | | | | | | | | | | |

**Supplementary Table S6: FREE2 DNA methylation in buccal epithelial cells of males with PM/FM mosaicism and comparison with control males**

|  |  | PM/FM | | | |  | Controls | | | | | |  | PM/FM vs Controls | |
| --- | --- | --- | --- | --- | --- | --- | --- | --- | --- | --- | --- | --- | --- | --- | --- |
|  | n | | Median (IQR) | Mean (SD)+ | Min-Max | | |  | n | Median (IQR) | Mean (SD) | Min-Max | |  | p-value* p-value** |
| MOR CpG1 | 8 | | 0.718 (0.220) | 0.706 (0.136) | 0.020-0.890 | | |  | 16 | 0.027 (0.038) | 0.039 (0.038) | 0.005 - 0.130 | |  | **0.0007 2.3x10^-14^** |
| MOR CpG2 | 8 | | 0.627 (0.240) | 0.606 (0.127) | 0.015-0.715 | | |  | 15 | 0.000 (0.010) | 0.009 (0.015) | 0.000 - 0.055 | |  | **0.0002 5.4x10^-14^** |
| MOR CpG6/7 | 8 | | 0.705 (0.140) | 0.698 (0.077) | 0.060-0.783 | | |  | 18 | 0.064 (0.045) | 0.070 (0.028) | 0.025 - 0.130 | |  | **0.0007 2.1x10^-19^** |
| MOR CpG8/9 | 8 | | 0.583 (0.172) | 0.601 (0.092) | 0.080-0.710 | | |  | 19 | 0.050 (0.020) | 0.052 (0.023) | 0.010 - 0.105 | |  | **0.0001 3.0x10^-18^** |
| MOR CpG10-12 | 8 | | 0.625 (0.218) | 0.614 (0.120) | 0.040-0.725 | | |  | 16 | 0.010 (0.006) | 0.015 (0.010) | 0.005 - 0.045 | |  | **0.0001 3.8x10^-15^** |

Abbreviations: n, number of participants’ samples from which MALDI-TOF MS results were available; IQR, interquartile range; SD, standard deviation Min – Max = minimum and maximum values; MOR, methylation output ratio; Adjusted p-values (for multiple testing using FDR) computed using *Mann-Whitney test to compare the median between the two groups and **two-sample t-test to compare the mean between the two groups, where +mean and SD in the PM/FM group computed with one outlier excluded; p-values less than 0.05 are highlighted in bold.
